# The History of Medicine and Surgery in Georgia

**Published:** 1895-02

**Authors:** Luther B. Grandy

**Affiliations:** Atlanta, Ga.


ATLANTA
### MEDICAL AND SURGICAL JOURNAL.

VOL. XI. FEBRUARY, 1895. No. 12.

EDITED BY
LUTHER B. GRANDY, M.D., AND MILLER B. HUTCHINS, M.D.,
WITH THE CO-OPERATION OF
H. V. M. MILLER, M.D., LL.D., VIRGIL O. HARDON, M.D.,
FLOYD W. McRAE, M.D., DUNBAR ROY, M.D.,
AND H. R. SLACK, M.D., PH.M.

### *ORIGINAL COMMUNICATIONS.*

### THE HISTORY OF MEDICINE AND SURGERY IN GEORGIA.*

BY LUTHER B. GRANDY, M.D.,
ATLANTA, GA.

One of the most prominent physicians of the State about this time was Dr. Richard Banks. Formerly he practiced at Ruckersville; in 1832 he moved to Gainesville. Dr. Banks had an immense practice, and his fame both as physician and surgeon was widespread. He was modest to a fault, and left no record of some of his best work. It is known, however, that he was the first American surgeon to extirpate the parotid gland. This was in 1831. The parotid region was then a *terra incognita* in surgery and the possibility of removing the gland was a mooted question. Dr. Banks's operation was done for a tumor of the parotid. To remove it and all of the diseased tissues it became necessary to dislocate the temporo-maxillary articulation. The operation is said to have been

*Continued from page 602, December number.

successful. But Dr. Banks's fame as a surgeon seems to have proceeded largely from his operations for cataract and for stone in the bladder. For cataract he operated by *couching*, a method no longer practiced. In lithotomy he was the contemporary and compeer of the celebrated Dudley of Kentucky. Their methods of operating were the same, and were those of their teachers in Philadelphia, Drs. Physick and Gibson. They did the lateral operation, and relied mainly on the *gorget* to get into the bladder. Up to about 1852 it is said that Dr. Banks had performed lithotomy sixty-four times, with but two unsuccessful results. He made no reports of his cases, and through fear of notoriety, he robbed himself of a reputation which would have been both great and just. Dr. Banks died in 1856. The present county of Banks was named in his honor.*

The medical act of 1825 did not require applicants to be examined who had a medical diploma from a lawfully chartered college. So, when this act was revived in 1839, the botanic doctors concluded that in their own defense a college must be had, primarily to confer diplomas, incidentally to teach and disseminate vegetable therapeutics, along with the latest and most approved methods of steaming, etc. So, the Southern Botanico-Medical College was organized at Forsyth. In 1846 the concern was moved to Macon, and in 1854 the name was changed to the Reform Medical College of Georgia.

There is nothing in the history of American medicine more replete with sad and tragic interest than the discovery of anesthesia. It seemed an irony of fate that this beneficent gift of God to man should become the *casus belli* for the bitterest controversy in our medical history—a controversy which, for some of the principals, led through disappointment and despair to insanity and suicide. It is of authentic record that the first discovery of anesthesia and the first surgical operation under ether were made in Jefferson, Jackson county, Ga., by Dr. Crawford W. Long. The story of that discovery, and of the circumstances which led up to it, has been told over and over again by Dr. Long and others. How he had learned of the intoxicating effect produced by inhaling the vapor of

**Atlanta Medical and Surgical Journal*, July, 1885.

sulphuric ether when a student at the University of Pennsylvania; how he used to administer it in sport to the young men of Jefferson who would assemble in his office to receive it; how he noticed that some of them would receive sprains and bruises in their intoxication, without complaining of pain until the effect of the ether had begun to wear off; how he reasoned from this observation that ether might be used to prevent pain in surgical operations; and how he determined to make the test as soon as occasion presented. All this has been told. His opportunity came, and his first operation was successfully made March 30, 1842, the removal of a small tumor from the neck of a Mr. Venable. Other operations were done, within the next three years, all of minor character, such as the amputation of fingers and toes, etc. Dr. Long anxiously desired an opportunity to test the anesthetic in capital surgery. He withheld his cases of painless minor surgery from precipitate publication because he wished to determine more accurately both the limitations and possibilities of ether. In his own words (*Southern Medical and Surgical Journal*, December, 1849): "I was anxious, before making my publication, to try etherization in a sufficient number of cases to fully satisfy my mind that anesthesia was produced by the ether, and was not the effect of the imagination, or due to any idiosyncrasy," etc. He then speaks of those "high in authority, who were advocating the mesmeric state as adequate to prevent pain in surgical operations. Notwithstanding thus sanctioned, I was an unbeliever in the science, and of the opinion that if the mesmeric state could be produced at all, it was only in those of strong imaginations and weak minds, and was to be ascribed solely to the patient's imagination. Entertaining this opinion, I was the more particular in my experiments in etherization."

Contemporary medical literature furnishes ample verification of these statements. The journals were full of discussions upon the phenomena of "mesmerism," "animal magnetism," etc., and wonderful reports were coming from European hospitals of surgical operations being done without pain during the "magnetic sleep." Among those who were especially active in investigating this subject were MM. Berna and Bertrand, of Paris; Elliotson, of London; Esdaile, of India; and in this country Professor J. K.

Mitchell, of the Jefferson Medical College, Philadelphia. Esdaile wrote a monograph entitled the "Practical Application of Mesmerism in Surgery." Oudet, of Paris, had extracted a tooth during the "magnetic sleep," and Jules Cloquet had excised a cancerous breast with the axillary glands, and the patients "showed no sign of pain" nor "uttered the least complaint."*

Topham, of London, in 1843, had amputated a thigh under the mesmeric influence. His patient, we are told, "felt no pain." In this country also there were those who claimed either to have performed or witnessed operations without pain during the magnetic state, and it was declared that "mesmerism is the *ne plus ultra* needed to kill pain in surgical operations." Among these was Dr. R. W. Gibbes, of South Carolina, an able and distinguished physician. To come still nearer home, Dr. L. A. Dugas, of Augusta, in January and November, 1845, had twice "extirpated the mamma of a female in the mesmeric sleep, without any evidence of sensibility during the operation." These operations were reported in the *Southern Medical and Surgical Journal,* March, 1845, and February, 1846. We can imagine the effect which these cases must have had upon the mind of Dr. Long, who believed that such results were to be ascribed solely to the imagination. Need we wonder, therefore, that he was the more particular in his experiments in etherization, wishing to try it in a sufficient number of cases to fully satisfy his mind that the anesthesia was produced by the ether, and not an effect of the imagination? Such were the reasons for his silence, and while the sequel was unfortunate, his course was cautious and commendable. Medicine then, as now, furnished many examples of the rise and fall of "discoveries" prematurely announced. He did not wish to add another. While thus waiting his opportunity was lost. A second discovery was made in Boston in 1846. The announcement was made as soon as it could be gotten into print. The discoverers patented their ether under the name of *letheon,* sold the "preparation" for twenty-five dollars per quart, and posted with precipitate dexterity to Congress for a reward of one hundred thousand dollars. Then came the celebrated ether controversy, which was terminated by preparations for war. After

---

* Transactions Royal Academy of Medicine, January, February, 1837.

the war the controversy was never reopened. One of the parties to it had died by his own hand; another had died insane; and another was confined to an asylum. Dr. Long was the fourth. He continued to practice his profession in Athens, beloved of everybody, until death came to him quietly in the midst of his labors. He died of apoplexy, June 16, 1878, while on a visit to a patient.

Dr. Long's original paper, presenting his claims, and reporting his cases, with certificates, etc., appeared in the *Southern Medical and Surgical Journal*, December, 1849. In May, 1877, Dr. Marion Sims again brought his case before the profession in an able paper in the Virginia *Medical Monthly*. Dr. Sims's paper contained some mistakes, more or less displeasing and unjust to Dr. Long. The present writer endeavored to correct these mistakes in an article in the Virginia *Medical Monthly*, October, 1893. The reader is also referred to a late book by Dr. L. W. Nevius, of New York, on the "History of the Discovery of Anesthesia," a fair and non-partisan statement of all the facts.

Dr. Tomlinson Fort, of Milledgeville, deserves more than the above passing references. Fifty years ago no physician in the State was more favorably known. He devoted himself faithfully to the duties of his profession, and we are told that he contributed his full share to the revolutions in the practice of medicine which occurred during his lifetime. Dr. Fort was opposed to the indiscriminate use of the lancet, and he was one of those who helped to deliver us from the reproach of a foreign writer that a "bloody Moloch presides in the chair of American medicine." He also pointed out the dangers of the too liberal use of mercurials, and assisted in reversing the irrational practice of prohibiting cold water to fever patients. In 1849 he published a volume of 700 pages, which he called a "Dissertation on the Practice of Medicine." This book was dedicated to the physicians of Georgia in acknowledgment of the kindness and confidence which the author had always received at their hands. It became popular, both with professional and lay readers. His description of diseases shows a thorough knowledge of his subject, besides giving the reader a clear insight into the high moral and professional attainments of the man.

Dr. Fort was successful in politics as well as in medicine. He was strictly a party man—an old Jackson democrat. He was in congress two years, and served Baldwin county eight years in the legislature. For thirty years (1825 to 1855) he took the liveliest interest in politics, both State and national; he "participated in the discussion of momentous questions with much crimination and re-crimination, with widespread hatred and life-long animosity, yet he came forth from it all in his old age universally beloved by all men of all parties. He stood through his long life above reproach."

Dr. Fort died in May, 1859, "at the summit of professional reputation, social standing, and political influence."*

Other well-known physicians of this time were: Dr. G. K. Holloway, of Warrenton; Dr. John Dent, of Augusta; Dr. P. M. Kollock, Dr. William R. Waring, of Savannah; Dr. Isaac Bowen, of Augusta; Dr. Edward Deloney, of Talbotton; Dr. F. M. Robertson, of Augusta; Dr. John B. Gorman, of Talbotton; Dr. Edward A. Eve, of Augusta; Dr. Richard D. Arnold, of Savannah; Dr. I. P. Garvin, of Waynesboro (and Augusta); Dr. G. F. Cooper, of Americus; Dr. H. M. Jeter, of Buena Vista; Dr. Thomas N. Hamilton, of Rome; Dr. C. B. Nottingham, of Macon; Dr. P. H. Wildman, of Savannah; Dr. E. M. Pendleton, of Sparta, and others.

In December, 1850, Dr. Jeter performed laparotomy for rupture of the uterus, and extracted a dead child and placenta from the abdominal cavity. Patient made a good recovery. (*So. Med. and Sur. Jour.*, March 1851.) Dr. Wildman introduced the tincture of the chloride of iron treatment for yellow fever. He gave 20–60 drops in water every two hours, and in the epidemic of 1854 he claimed to have thus treated 150 cases without a death. He gave no other medicine. Dr. Wildman has the greatest faith in this remedy, regarding it as both curative and prophylactic. He himself fell a victim to the disease during the epidemic. Among those who began their careers in Georgia during this period and afterwards achieved their reputations in other States were, Drs. John and Joseph LeConte (University of Georgia and University of California), well known from their works in natural science, and Dr.

*Atlanta Medical and Surgical Journal*, June, 1885.

Joseph Jones, now the distinguished professor of chemistry and medical jurisprudence in Tulane University, New Orleans.

To Dr. Lewis D. Ford we owe much of our present knowledge as to the nature and treatment of the malarial fevers. His papers on this subject are to be found in the *Southern Medical and Surgical Journal*, 1836 to 1847. Dr. Ford sought to establish the true relationship existing between " malignant intermittent," " intermittent," and " remittent " fevers; and in opposition to prevailing doctrines, he proceeded to show that " though different in many of their external features, yet they [were] fundamentally of the same nature and required the same mode of treatment." This mode of treatment, according to Dr. Ford, was the " abortive plan," by large doses of quinine—large enough to prevent a recurrence of the paroxysms.

It was the current teaching and practice that these diseases must run their course of paroxysms and remissions, and quinine was only mentioned as an adjuvant to the old routine methods by cathartics, bleeding, mercurials, blisters, bark and wine, etc. Dr. Ford's practice was to give ten or fifteen grains of quinine every two hours until about forty grains were taken. With the spread of this teaching and under this treatment intermittent fever ceased to be the " despair of organic medicine."

Dr. Ford was the Nestor of medical education in Georgia. For nearly fifty years he was Professor of the Principles and Practice of Medicine in the Medical College of Georgia, and Dean of the college for many years. His unsuccessful effort to convene an association of medical colleges in 1835, to devise ways and means for improving the status of medical education, has already been referred to. Dr. Ford was the first president of the Medical Association of Georgia (Macon, 1849).

Dr. Paul F. Eve was a distinguished Southern surgeon, and no one was ever held in higher professional esteem. He was one of the founders of the Medical College of Georgia, and for eighteen years professor of surgery. The volumes of the *Southern Medical and Surgical Journal* contain many important papers by Dr. Eve. Among these should be mentioned his series of "Surgical Cases" 1836–1838); " Lecture on Mesmerism," delivered at the request of the students, February, 1845. This is a complete and admirable presentation of this whole subject, *pro* and *con.* Dr. Eve did not accept the evidence in favor of the surgical uses of mesmerism, or animal magnetism. He believed that all reported cases could be explained as effects of the imagination alone.

In April, 1854, Dr. Eve introduced in America the bilateral operation of lithotomy. His first paper appeared in the *American Journal of Medical Sciences*, of above date, reporting four cases. This was Dr. Eve's favorite method of operating for stone, and he did more than any other American surgeon to give this operation a recognized status in this country. The first successful abdominal hysterectomy in America was performed by Dr. Eve in April, 1850. This was done for malignant disease. The patient lived four months and died from a recurrence of the growth.

Other important papers up to this time of his life were: "Lithotrity and Lithotripsy in the United States;" "Lithotomy—117 Calculi Successfully Removed;" "Chloroform and Operations under its Influence;" "Operations on the Jaws, with Results in Fourteen Cases" (reporting several cases of removal of the superior and inferior maxilla, in whole or in part); "Reply to Dr. Oliver Wendell Holmes on our National Medical Literature" (a very interesting discussion); "Unsuccessful Cases in Surgery" (a candid and honest record of some important cases occurring in his practice, having an unfavorable issue).

For a number of years (1845–1849) Dr. Eve was editor of the *Southern Medical and Surgical Journal*, conducting it on a high plane and with marked ability. In November, 1850, he was called to the chair of surgery in the University of Louisville. Afterwards he was professor of surgery in the University of Nashville. His "Address on (American) Surgery" at the International Medical Congress, Philadelphia, 1876, was a scholarly effort, possibly the best of his lifetime.

The Georgia Medical Association had its origin in a meeting of physicians in Macon, March 20, 1849. This meeting was called by the Medical College of Georgia and the local medical societies of Savannah and Macon. About eighty delegates were present. Following officers were elected: President, Dr. Lewis D. Ford,

Augusta; First Vice-President, Dr. R. D. Arnold, Savannah; Second Vice-President, Dr. Thomas R. Lamar, Macon; Corresponding Secretary, Dr. J. M. Green, Macon; Recording Secretary, Dr. Charles T. Quintard, Macon. Aside from organization about the only business transacted at this meeting was the appointment of a committee of five to memorialize the legislature on the necessity of instituting a regular registration of births, marriages, and deaths in the State. Of the eighty charter members of the Society only Dr. J. F. Alexander of Atlanta, and Dr. S. D. Brantley of Sandersville, are now living (January, 1895).

Dr Joseph A. Eve was another ornament to the Georgia profession. For a number of years he was the associate of Dr. Antony, and from 1832–1839 he was professor of Materia Medica and Therapeutics in the Medical College of Georgia. At the death of Dr. Antony he was transferred to the chair of obstetrics and diseases of women, which he filled with distinction and ability until his resignation in 1885. His favorite and especial line of practice was obstetrics and gynecology, and in this field his work was always marked by a conscientious conservatism.

He was an excellent teacher, and his lectures and papers have done much to mould the past and present generations of Southern physicians. At the time of his resignation from the college he was the oldest teacher of obstetrics in the world. Dr. Eve co-operated heartily with Dr. Ford in the latter's efforts in behalf of a better medical education. In September, 1836, Dr. Eve published (*So. Med. and Sur. Jour.*) an able article which emphasized the "four cardinal defects in the present system of medical education in the United States, viz.: the want of preparatory learning as a requisite to matriculation; the paucity of branches taught, the shortness of each session of lectures, and of the whole peried of collegiate instruction before graduation." His paper was a complete and irrefutable answer to those who were saying that the time had not yet come for the proposed reforms in medical education. But his paper and the appeal of Dr. Ford for a convention of colleges came and went like the voice of one crying in the wilderness.

Dr. Eve was editor of the *Southern Medical and Surgical Journal* for several years, and most of his contributions to medical literature are to be found here. Some of these were: "The Modus Operandi of Medicines," "Professional Qualifications and Character;" "Debilitants and Sedatives;" "Remarks on Secale Cornutum (Ergot) in Obstetric Practice," in which the author advises a more cautious use of the drug, and points out its proper indications; "Convulsions and other Nervous Affections During Pregnancy and the Puerperal State;" "The Use of Chloroform in Obstetric Practice;" "Clinical Contributions to Obstetrics and Gynecology."

Dr. Eve was president of the Georgia Medical Association in 1878. He died in 1886, having labored in his profession for more than sixty years.

The name of Dr. Henry F. Campbell, of Augusta, has been an honor to the profession of Georgia and to American medicine. He graduated at the Medical College of Georgia in 1842, and in the fifty years following served the college with distinction and ability in the Chairs of Anatomy, Surgery, and Gynecology. For two years of this time (1866–67) he occupied the Chairs of Anatomy and Surgery in the New Orleans School of Medicine.

Dr. Campbell was the first to practice and advocate the ligature of the main artery going to a part for the treatment of inflammation and threatened gangrene. Arterial compression had been advised for this purpose, but this necessitated some interference with the venous circulation, a clear disadvantage. So, tying the main artery was recommended by Dr. Campbell. He operated with success in a number of cases, chiefly for gunshot injuries of bones and joints during service in war. (See Confederate Manual of Military Surgery, Richmond, 1863, page 297; also article by Dr. Campbell, "The Hunterian Ligation of Arteries in Destructive Inflammation," *Southern Journal of Medical Sciences*, New Orleans, August, 1866.)

Dr. Campbell's several papers relating to the excito-secretory and excito-motor system of sympathetic nerves represent some of his best work. His first appeared in the *Southern Medical and Surgical Journal*, June, 1850, in connection with the "The Influence of Dentition in Producing Disease."

Within the next two or three years the same system of nerves was independently discovered and described by both Dr. Marshall

Hall of London, and M. Claude Bernard of Paris. Upon learning of Dr. Campbell's studies and paper Dr. Hall promptly awarded him the credit of priority, saying: "It would be unjust to deny that Dr. Campbell has the merit of having first called attention to the excito-secretory system, in the year 1850, and that he imposed this very designation in 1853."

The following papers by Dr. Campbell completed this series: "The Sympathetic Nerve in Reflex Phenomena—a Question of Priority of Announcement with M. Claude Bernard" (Transactions American Medical Association, 1853); "The Excito-secretory System of Nerves," the prize essay at the American Medical Association, 1857. The discussion with Dr. Marshall Hall concerning the "Priority of Announcement in Reflex Secretion and the Excito-secretory System of Nerves" will be found in the *Southern Medical and Surgical Journal*, 1857, and London *Lancet*, May 2, 1857.

Dr. Campbell offered the most rational explanation of the action of quinine on the parturient uterus, from its properties as a cerebro-spinal depressant.* He showed (Transactions American Gynecological Society, Vol. V) that a constant existence of pain exercises an obtunding influence over nervous reflexes, and that in parturition such a condition would retard the progress of delivery; at this point quinine acts by "obtunding the sensibility of the inhibitory centers of the cord, allows the normal reflexes to go on, and so indirectly increases the action of the uterus."

Dr. Campbell was the first to utilize and advise the knee-chest posture in the treatment of uterine malpositions. (See "Position, Pneumatic Pressure, etc., in Uterine Displacements," *Atlanta Medical and Surgical Journal*, June, 1875; also Transactions American Gynecological Society, 1875.)

Other important papers by Dr. Campbell were: "The Nature of Typhoidal Fevers," "Bilateral Lithotomy," "The Georgia Military Hospitals of Richmond," "Blood-letting in Puerperal Eclampsia," "Urinary Calculi" (Transactions American Medical Association, 1879). He was elected president of the American Medical Associ-

*Petijean had asserted in 1846 that quinine possessed oxytocic properties, and Dr. J. S. Wilson, of Lawrenceville, Ga., had called attention (*So. Med. and Surg. Jour., June*, 1855) to the action of quinine in increasing the menstrual and lochial discharges, suggesting further, "a more direct, immediate, and specific effect on the uterus.")

ation in 1885. He was president of the Georgia Medical Association in 1871, and in 1876 one of the founders of the American Gynecological Society.

(*To be concluded.*)

